# Recent Reviews of Syphilis

**Published:** 1917-01

**Authors:** B. F. Stout

**Affiliations:** San Antonio, Texas


### CLINICAL PATHOLOGY

Dr. B. F. Stout, Editor, 203 Hicks Bldg., San Antonio, Texas

Communications solicited and should be sent direct to Dr. Stout. Copy should be in Dr. Stout's possession by the 20th of the preceding month.

### Recent Reviews of Syphilis.

BY B. F. STOUT, M. D., SAN ANTONIO, TEXAS.

The past few years have witnessed a most remarkable advance in our knowledge of the pathology of syphilis owing to the discovery of the causative organism, its cultivation later by Noguchi, and the diagnosis of the disease by means of the Wassermann reaction and the Luetin test. The mass of literature on the subject is enormous and few are in a position to be able to keep up with it as they would wish. For the benefit of those who desire to bring themselves up to date the writer begs to call attention to a series of articles in the October number of the *American Journal of the Medical Sciences.* In this number are discussions by such authorities as Fordyce, Homer Swift, Warthin and Veeder. It will well repay anyone to read these articles carefully.

For those who feel they have not the time or have not access to this journal the more important points will be set forth here. Diagnosis: Fordyce points out under this heading the importance of early diagnosis, particularly calling attention to the danger of overlooking the presence of the disease in what appear to be ordinary soft sores. It is well known that these frequently harbor the parasite of syphilis, and it is growing to be more and more the opinion of the specialists in venereal disease that there is no certain way of distinguishing between chancre and chancroid except by careful search for the spirochete, best by dark field illumination.

Here the writer of this article wishes to mention a point not brought out by Fordyce, and that is the desirability of not using any cauterizing application to the sore before making the examination for the spirochete. Syphilis can be diagnosed within a few days after the sore has appeared by this means, and much valuable time saved to the patient. Every day means a more widespread systemic infection with the chance of a cure correspondingly less. After the disease has existed for some time, diagnosis must depend largely on the Wassermann test of the blood and spinal fluid and on the various other tests of the spinal fluid such as the cell count, globulin tests and the colloidal gold reaction of Lange. This last test is one easy of application, but rendered less available because of the very great care necessary in preparing the reagent.

Few who have not studied the pathology of syphilis in a special way realized the importance of the spinal fluid examination. One is astonished to learn, according to Swift, that the central nervous system is involved in the secondary stage of syphilis in from 66 per cent to 90 per cent of the cases. Many cases of advanced disease, showing a positive blood Wassermann in spite of vigorous treatment will show involvement of the central nervous system by means of spinal fluid tests. By these tests we are able to determine not only the presence and severity of the syphilitic process but can distinguish between the various types. The effect of treatment may also be very accurately observed by this method. As a matter of fact, it is becoming the general opinion of those best informed that no patient should be considered cured of his disease without a normal spinal fluid as well as a Wassermann negative blood.

It has been the writer's experience that many patients are given a short course of salvarsan and mercury or go to Hot Springs and are given the baths and rubs there for a short time and then are assured they are cured without any tests of blood or spinal fluid. Many turn up later with serious recurrences and of course not a few with incurable lesions.

It is not within the province of this department to go into the matter of treatment, important as it is, but the necessity of long and carefully controlled treatment must appeal to all who think deeply on this subject. Swift who in his article deals wholly with the nervous system calls very particular attention to the prevention of the development of paresis and tabes by making very early diagnosis. The recognition of the monosymptomatic forms of syphilis of the nervous system is necessary in this connection. Among these is Erb's ophthalmoplegia interna, which has been followed by fatal paresis.

Transitory squint may be present for years before the development of tabes.

Surgeons frequently see early manifestations in abdominal diseases. Nuzum (*J. A. M. A.*, 1916, Volume LXVI, 482) recently reported that, among a thousand cases of tabes admitted to Cook County Hospital, 87 had undergone operation because of mistaken diagnosis of their pain. Swift says that abdominal pain may be present for years before the full development of tabes. Other conditions are bladder disturbances, leg pains and so-called "rheumatism." Where these symptoms persist, Wassermann tests of blood and spinal fluid should always be made.

Warthin (Professor of Pathology at Ann Arbor) writes most interestingly of the persistence of active lesions and spirochetes in the tissues of clinically inactive or "cured" syphilis. This author finds of 41 cases in which syphilis was demonstrated at autopsy that 11 were regarded as cured, and 25 were not suspected at all of having the disease. He was very pessimistic as to cure in this disease and does not believe that the reported cases of reinfection are above suspicion.

Regarding this matter the writer differs with Dr. Warthin, having recently seen a case which must be regarded as one of reinfection. This patient was infected five years ago, gave a positive Wassermann and classical symptoms, was vigorously treated for three years and for two years has given repeated negative Wassermanns. Six weeks ago he came to the writer with a small sore, not at the site of the old one. Four weeks later he developed a typical secondary rash with a four plus positive Wassermann reaction. He admitted exposure two weeks before the primary lesion appeared. It is hard to explain this case other than one of reinfection.

However, one must never allow themselves to be lulled to a sense of false security by a negative Wassermann or a series of negative tests. The writer always advises his patients to have tests made at least once a year for many years after apparent cure has resulted, since latent syphilis does not give any sign of its presence.

As Warthin says latent syphilis is of the greatest sociological importance, being upon a plane, almost equaling tuberculosis as a factor opposed to the health and progress of the race.

---

No physician can afford to be without his "sign." The Globe Metal Sign Works manufacture brass or bronze tablets for the profession. Their signs are beautiful and artistic, and reasonable in price. They would like to show you just how superior they are.

